# Phytochemical and Antioxidant-Related Investigations on Bark of *Abies spectabilis* (D. Don) Spach. from Nepal

**DOI:** 10.3390/molecules17021686

**Published:** 2012-02-08

**Authors:** Stefano Dall’Acqua, Paola Minesso, Bharat Babu Shresta, Stefano Comai, Pramod Kumar Jha, Mohan Bikram Gewali, Emanuela Greco, Rinaldo Cervellati, Gabbriella Innocenti

**Affiliations:** 1 Department of Pharmaceutical Sciences, University of Padova, Via Marzolo 5, Padova 35121, Italy; 2 Central Department of Botany, Tribhuvan University, Kirtipur, Kathmandu 44600, Nepal; 3 Central Department of Chemistry, Tribhuvan University, Kirtipur, Kathmandu 44600, Nepal; 4 Department of Chemistry “G. Ciamician”, University of Bologna, Bologna 40126, Italy

**Keywords:** *Abies spectabilis*, HPLC-MS/MS, NMR, phenols, antioxidant activity

## Abstract

The bark of several coniferous species, a waste product of the timber industry, contains significant amounts of natural antioxidants. In our ongoing studies of Nepalese medicinal plants, we examined the bark from *Abies spectabilis* as the starting material for extracting antioxidant compounds. *In vitro* antioxidant activity evaluated by means of three antioxidant methods, namely 2,2-diphenyl-1-picrylhydrazyl (DPPH), Briggs-Rauscher oscillating reaction (BR) and Trolox Equivalent Antioxidant Capacity (TEAC) and total phenol contents with the Folin-Ciocalteau reagent; the ferrous iron chelating capacity was also assessed. The methanol extract of *A. spectabilis* showed significant antioxidant activity and polyphenol contents (IC_50_ 4.13 µg/mL, 0.20 μg/mL eq. resorcinol, 4.22 mM eq. Trolox, 3.9 µg/g eq. gallic Acid in the DPPH, BR, TEAC and Folin-Ciocalteau tests, respectively) and weak Fe^2+^ chelating capacity. Phytochemical studies were also carried out with 1D- and 2D NMR experiments and DI-ESI-MS, HPLC-DAD and LC-MSn measurements. Oligomeric C-type proanthocyanidins, mainly trimeric gallocatechin derivatives, were the most abundant compounds (16% of extract expressed as procyanindin B1). Gallocatechin oligomers (up to six units) and prodelphynidin-gallocatechin polymers were also identified in the extract. Prodelphynidin B4, cyclograndisolide and trans-docosanil ferulate were also isolated and characterized by NMR and MS spectroscopy.

## 1. Introduction

Although the bark of coniferous tree species is considered as a waste product in the timber industry, it is rich in secondary metabolites, especially phenol and lignan derivatives [1,2,3,4]. As an example, a standardised extract from the French maritime pine (*Pinus pinaster*) bark has been patented as Pycnogenol®, which is used extensively in the cosmetics and herbal products industry [5,6,7,8,9,10]. 

There is great interest in the development of new naturally derived active ingredients for cosmetic, nutraceutical, functional food and pharmaceutical products [11,12], and Nepalese medicinal plants are a quite unexplored source of potentially useful secondary metabolites. Nepal, by virtue of the fact that, it rises almost from sea level (ca. 70 m a.s.l.) to Mt. Everest, the highest mountain peak in the world (8,848 m), presents rich and varied plant and animal biodiversity [13]. In addition, this great biodiversity, as well as abnormal and harsh growing conditions for plant species in several areas (e.g., at high altitudes) result in great chemical diversity in secondary metabolites. For some years now, we have been studying the chemistry and biology of some Nepalese medicinal plants such as *Curculigo orchioides*, *Lindera neesiana*, *Aconitum naviculare* and *Rhododendron anthopogon* [14,15,16,17,18]. In the present study, we examined the bark of *Abies spectabilis* (Pinaceae). This plant is a evergreen coniferous tree about 50 mt high, distributed throughout Nepal at 2,400–4,200 m a.s.l. in moist, open areas; it is also distributed in India and Bhutan. This fir has many uses in the traditional medicine of several mountain areas in Nepal. For example, in the Manang district (2,000–6,000 m a.s.l.), a paste made of the leaves and cones of *A. spectabilis* is externally used to treat fractures, although there is no information about the traditional uses of *A. spectabilis* bark. Information about the phytochemical composition of this material is lacking. Extracts obtained from the bark were examined for their *in vitro* antioxidant activity, and phytochemical fingerprints were also obtained by both non-chromatographic (NMR and MS) and chromatographic (HPLC-DAD, HPLC-MS/MS) methods.

## 2. Results and Discussion

### 2.1. Antioxidant Activity and Iron (II) Chelating Capacity

The antioxidant activity of the methanol *A. spectabilis* bark extract (ME) was remarkably high, the IC_50_ for the 2,2-diphenyl-1-picrylhydrazyl (DPPH) test (4.13 ± 0.02 μg/mL) being comparable to the reference compounds rutin (4.80 ± 0.02 μg/mL) and ascorbic acid (3.50 ± 0.03 μg/mL) (see Table 1). The values obtained with the Briggs-Rauscher oscillating reaction (BR) method (0.20 ± 0.02 μg/mL eq. resocinol) and Trolox Equivalent Antioxidant Capacity (TEAC) assay (4.2 ± 0.2 mM eq. Tr) were also far higher than those reported for extracts of several plants used in folk medicine [19]. In addition, the Folin-Ciocalteau (FC) reagent revealed a Gallic Acid Equivalents (GAE) value of 3.9 ± 0.1 μg/g, higher than those of other medicinal plant extracts [19]. The FC method suffers from the effects of several interfering substances present in plants, as the FC reagent oxidizes not only phenolics, but also a number of other substances, such as ascorbic acid, sulphur-containing compounds, mono- and disaccharides that are naturally present in vegetables [20,21,22], but despite its limitations for quantifying phenol compounds in complex matrices, the FC method is still recommended for measuring total reducing capacity [20,21]. The reducing capacity of a sample is an important parameter, reflecting one aspect of its antioxidation ability, because reduction is involved in maintaining cellular redox status, in signal transduction and metal cycling, and also may have pro-oxidant effects [20,21,22].

**Table 1:** Antioxidant activity of *A. spectabilis* bark extracts. Ascorbic acid and rutin were used as reference compounds.

Extract	DPPH IC_50_ μg/mL	GAE μg/g
Methanol	4.13 ± 0.02	3.9 ± 0.1
Chloroform	>100	n.d.
Rutin	4.80 ± 0.02	n.d.
Ascorbic acid	3.50 ± 0.01	n.d.

n.d.: not determined.

DPPH and FC tests on the chloroform extract revealed poor activity. These results are not unexpected due to the poor solubility of polyphenol derivatives in lipophylic solvents. Ferrozine can quantitatively form complexes with Fe^2+^ and, in the presence of other chelating agents, complex formation is inhibited and the red colour of the complex is decreased. For this reason, determination of the reduction of the colour allows estimation of the chelating activity of the sample in question. The scavenging of transition metals is important because of their ability to catalyse oxidative reactions and thus, the capacity of extracts to chelate metals is a significant antioxidant-related property [23]. Our *A*. *spectabilis* extracts showed little ability to chelate iron(II) and did so in a concentration-dependent fashion. However, when compared with the standard compound EDTA (EC_50_ 0.27 mg/mL), the *A*. *spectabilis* extract presented 10% Fe^2+^ chelation at 3.5 mg/mL. These results match previously published data for *Pinus sibirica*, suggesting that this activity is not useful in protecting against Fenton chemistry/hydroperoxide decomposition-mediated oxidative damage *in vivo* [24].

### 2.2. Phytochemical Composition of Methanol Extract

**Figure 1:**
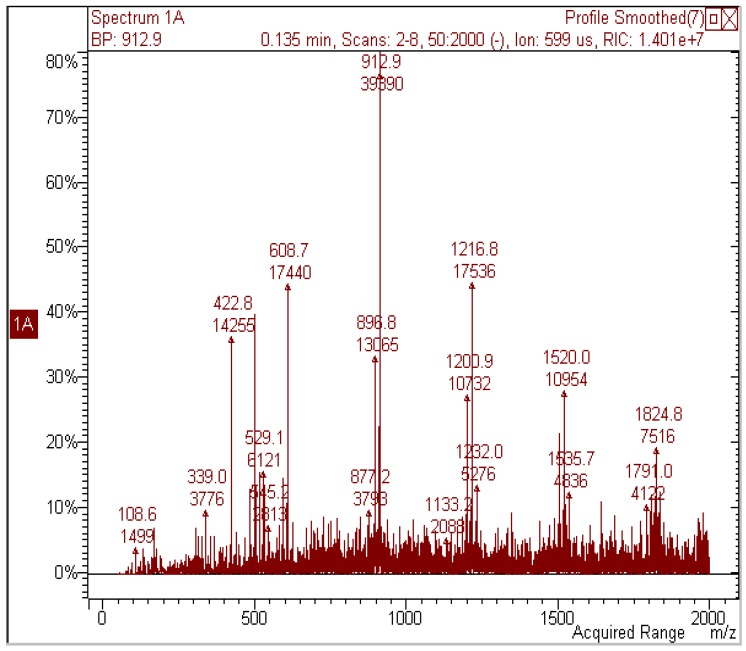
Mass spectrum of the methanol *A. spectabilis* bark extract.

The ^1^H-NMR of the *A. spectabilis* methanol extract (ME) was very crowded (see Supporting Information) and several aromatic proton signals were detected in the region of δ 7.35–5.60. In the region at δ 4.80–3.30, peaks were crowded, due to several aliphatic groups linked to electronegative atoms supporting sugar portions and carbohydrate structures. The HSQC-DEPT spectrum (see Supporting Information) was recorded in order to link proton signals with their counterpart carbon atoms. Several signals supporting the presence of gallocatechin units [3,25,26,27] were observed, *i.e.*, proton signals in the region δ 4.21–4.30, linked to carbon resonances at δ 82.5, 82.7, 82.4, 71.7, 72.1 and 71.0, ascribable both to sugar portions and the C-3 and C-2 positions of gallocatechin moieties. Further correlations were observed from different proton signals at δ 4.48–4.51 with carbon resonances at δ 37.7–37.2 and from a proton signal at δ 2.84 and a carbon resonance at δ 28.7. These data are compatible with the C-4 of gallocatechin moieties, as previously reported [3,29,30,31]. All these preliminary NMR findings suggested that the methanol extract of *A. spectabilis* bark contained gallocatechin derivatives. To confirm our initial NMR findings, we subjected *A. spectabilis* methanol extract to DI-ESI-MS measurements in negative ion mode. The main ion species detected were [M-H]^−^ at *m/z* 423, 593, 609, 897, 913, 1217, 1520 and 1825 (see Figure 1). Many of these species are in multiples of 305, suggesting various oligomeric species. The isolation of each species in the ion trap and further MS/MS analysis yielded the characteristic fragmentation pattern of proantocyanidins. [28]. The main ions of the extract and their MS/MS fragments are listed in Table 2. MS spectra due to Retro Diels Alder (RDA) and heterocyclic ring fission (HRF) fragments allowed us to identify dimers (*m/z* 609), trimers (*m/z* 913), tetramers (*m/z* 1215), pentamers (*m/z* 1536) and hexamers (*m/z* 1825). MS/MS analysis of the ion at *m/z* 593 produced a fragment at *m/z* 441, corresponding to a mass loss of 152 Da (RDA) and indicating that the upper unit of this heterodimer was 3’,4’-dihydroxyflavan-3-ol [28]. The spectra and fragmentation patterns of the three main species are given in the supporting information.

As previously indicated by NMR data, DI-ESI-MS experiments confirmed the presence in the extract of proantocyanidin derivatives, mainly gallocatechin polymers. We modified the method used for cocoa proantocianidine derivative analysis, which employs hydrophilic interaction liquid chromatography (HILIC) with a diol stationary phase [29]. Instead of using the fluorometric detector, we modified this method by coupling the HPLC with an MS ion trap and DAD.

**Table 2:** Main [M−H]^−^ ions observed in the DI-ESI spectrum and their collisionally generated fragments. Identification of compound structures was achieved on the basis of fragmentation pattern and comparison with literature data. Letters are indicating catechin unit (C), gallocatechin unit (G), upper units in the dimeric/polymeric derivatives are the first letter.

[M−H]^−^ *m/z*	MS^2^	MS^3^ (parent ion)	Proposed structure
593	467; 441; 423; 305	(467) 305; (441) 423	G-C
609	591; 483; 441; 423; 305	(483) 305; (441) 423	G-G
897	771; 729 ; 711	(771) 593; (729) 711	G-C-G
913	787; 745; 609	(787) 609, 483; (745) 727	G-G-G
1217	1091; 1049; 305	(1091) 913; (1049) 1031	G-G-G-G

**Figure 2:**
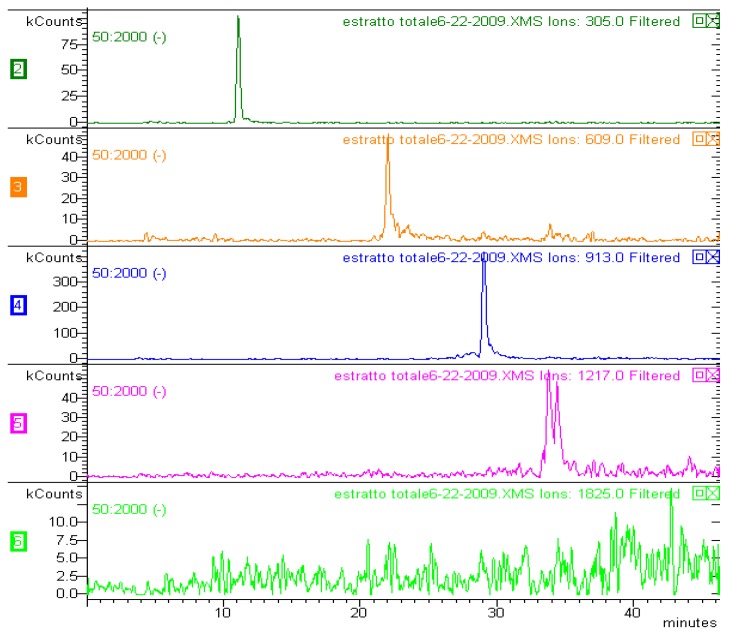
LC-MS chromatogram of the methanol *A. spectabilis* bark extract.

The HPLC-MS results shown in Figure 2 for the HILIC analysis show that the proantocyanidin derivatives are eluted at increasing retention times, according to the extent of polymerisation. Gallocatechin oligomers (up to six units) and prodelphynidin-gallocatechin polymers were identified in the extract, matching results from NMR and DI-ESI-MS without prior chromatographic separation.

**Figure 3:**
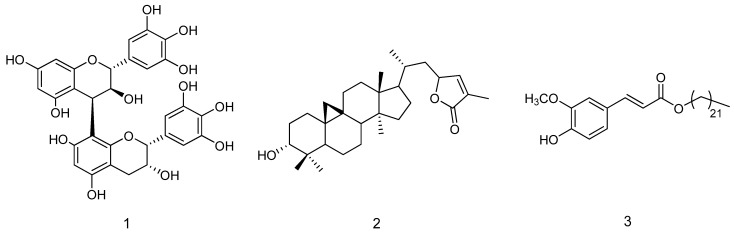
Chemical structures of compounds isolated from the bark extract.

We also estimated the amount of proantocyanidin in the extract, by HPLC-DAD and procyanidin B1 as a standard compound. Proantocyanidin content was 16.6%, expressed as procyandin B1. Phytochemical investigations on the ME extract yield in the isolation of prodelphynidin B4 (**1**) The structure of the isolated compounds (Figure 3) was elucidated on the basis of extensive 1D and 2D NMR experiments and MS spectroscopy, and by comparison with literature data [2,27,28].

### 2.3. Phytochemical Investigation of Chloroform Extract

In order to evaluate lipophilic compounds in the extracts, the chloroform fraction (CH) was also analysed. The ^1^H-NMR spectrum revealed an extremely crowded aliphatic region, although a triplet signal at δ 2.34 was detected. A large broad singlet at δ 1.25 and a triplet at δ 2.34 indicated fatty acid hydrocarbon chains. Resonances in the region δ 4.00–4.30 also supported the presence of glycerol or aliphatic alcohols linked to fatty acids. Doublets at δ 6.28 and 7.55 (*J* = 15.5) supported the presence of *trans* olefinic double bonds. The great difference in their chemical shift (Δ = 1.27 ppm), the deshielded value of one of the protons (δ 7.55) and the presence of other characteristic aromatic signals at δ 7.06 and 6.82 allowed us to identify a phenylpropanoid unit with a carbonyl function conjugated to the olefin moiety.

Further phytochemical investigations on the chloroform extract afforded cyclograndisolide (**2**) and *trans*-docosanil ferulate (**3**) (see Figure 3). The structure of isolated compounds were elucidated on the basis of extensive 1D and 2D NMR experiments and MS spectroscopy, and by comparison of their spectral data with literature results [30,31,32].

## 3. Experimental

### 3.1. Chemicals

Malonic acid, manganese (II) sulphate monohydrate, NaIO_3_, Na_2_CO_3_ anhydrous, resorcinol (1,3-benzenediol) (all reagent grade >99%) were purchased from Merck. Gallic acid (3,4,5-trihydroxy benzoic acid,), 2,6-DHBA (2,6-dihydroxybenzoic acid), Trolox (6-hydroxy-2,5,8-tetramethylchroman-2-carboxylic acid), FeCl_2_, and ferrozine from Sigma Aldrich; K_2_S_2_O_8_, 2,2′-azino-bis-(3-ethylbenzthiazoline-6-sulphonic acid) (ABTS), Folin–Ciocalteau reagent (FC), 2,2-diphenyl-1-picrylhydrazyl (DPPH), HClO_4_ and H_2_O_2_ were purchased from Fluka. HClO_4_ was analysed by titration *vs.* a standard 0.1 M NaOH solution from Merck. H_2_O_2_ was standardised daily by manganometric analysis. All stock solutions were prepared with double distilled (dd), deionised water.

For chromatographic separation, Merck 60 F_254_ (cod. 5715) silica gel plates and Sephadex LH-20 (Sigma-Aldrich) were used. Deuterated, pure and analytical grade solvents were obtained from Sigma-Aldrich.

### 3.2. General Experimental Procedures

NMR spectra in CD_3_OD were obtained on a Bruker AMX-400 spectrometer, operating at 400 MHz for ^1^H-NMR and 100 MHz for ^13^C-NMR. 2D experiments, ^1^H-^1^H DQF-COSY, NOESY, TOCSY and inverse-detected ^1^H-^13^C HSQC spectra were performed with TOPSPIN software. 

The LC-MS equipment (Varian) comprised a chromatographic system (Varian LC-212) coupled with a Varian 500-MS (ion trap) mass spectrometer fitted with an ESI source (Varian). The MS conditions were the following: Needle voltage, −4500 V; nebulising gas, nitrogen; nebulising gas pressure, 1.72 bar; drying gas pressure, 1.03 bar; drying gas temperature, 400 °C; capillary voltage, 100 V; RF loading, 75%; MS range, 50–2,000 Da. Parameters were optimised with the Varian WS workstation by directly injecting 10 µL/min solutions of proantocyanidin B1 (1 µM). MS^n ^spectra were obtained both in direct injection mode and during chromatographic runs by means of the turbo-dds (tdds) function of the instrument. The LC-MS equipment was used for direct injection into the ion trap mass spectrometer (DI-IT-MS) and for HPLC-MS^n^ analyses. Extracts were dissolved in methanol and solutions were directly injected into the IT spectrometer at a rate of 10 µL/min.

HPLC-DAD was performed on an Agilent 1100 series liquid chromatograph equipped with a diode array UV-Vis detector (1100 series) with an Agilent LiChrosphere® 100 Diol (4 × 250 mm, 5 µm) column and acetonitrile/acetic acid 98:2 (solvent A) and methanol/water/acetic acid 95:3:2 (solvent B) as eluents. Gradient eluition started from 100% (A), in 35 minutes 60% (A), 40 minutes 60% (A) and then 45 minutes 100% (A). The flow rate was 600 µL/min and the injection volume 20 µL. Before injection into the MS spectrometer, the flow was split into two parts, allowing to introduction into the MS spectrometer of 300 µL/min. Procyanidin B1 (Phytolab GmbH) was used as reference compound, and was detected at 280 nm. Calibration curve was prepared with the standard sample at four concentration levels, ranging from 5.5 to 109.5 μg/mL. LOQ and LOD were 7.5 μg/mL and 2.5 μg/mL, respectively. Peaks corresponding to proanthocyanidins were identified according to their UV spectra. 

### 3.3. Plant Material

*A. spectabilis* bark was collected in October 2007 from Sagarmatha National Park (Nepal) at an altitude of 3,300 m, and was authenticated by one of the authors (B.B.S.). A voucher specimen (No. AS07) was deposited at the Department of Pharmaceutical Sciences, University of Padova. 

### 3.4. Extraction and Isolation

*A. spectabilis* bark (50 g) was ground and defatted with petroleum ether and then extracted with methanol (250 mL) at room temperature in an ultrasound bath to give the ME extract. Extraction was repeated three times. The solvent was collected and removed under vacuum, yielding a solid brown residue (7% yield). Part of the residue (2 g) was dissolved in a methanol/water mixture 1:10 and then partitioned with chloroform (CH). The organic layer was dried with sodium sulphate and the solvent was removed under vacuum, yielding residue (1.5%). The ME extract was applied to a Sephadex LH-20 column and eluted with a water/acetone mixture (60:40). Fractions were pooled according to their TLC and mass (direct injection in ion trap mass spectrometer) characteristics. Further chromatographic purification on Sephadex LH-20 with acetone 40% yielded compounds **1** (7.0 mg). The CH extract on repeated preparative TLC with a mixture of cyclohexane/ethyl acetate (2:1) and dichlorometane/methanol (90:10), afforded compounds **2** (4.0 mg) and **3** (12.0 mg).

### 3.5. Antioxidant Activity

#### 3.5.1. DPPH Assay

The scavenging activity towards the 2,2 diphenyl-2-picrylhydrazyl (DPPH) radical was measured as previously described [19]. Stock solutions (1–3 mg/mL) of the extracts were prepared and added to 30 μg/mL methanol solutions of DPPH to reach final concentrations of sample of 0–200 μg/mL. Mixtures were kept at room temperature for 30 minutes in the dark, and absorbance at 517 nm was then measured against a blank solution at the same DPPH concentration but without the extract. For the ME extract, five or six dilutions were measured in triplicate, a linear range of concentration *vs.* % decrease of absorbance was observed and consequently used to determine the IC_50_. Rutin and ascorbic acid were used as standard compounds. 

#### 3.5.2. BR (Briggs-Rauscher Oscillating Reaction) Method

The chemical *in vitro* method previously reported by Cervellati *et al.* [33], is based on the inhibitory effects of free radical scavengers on oscillations in the BR reaction. In brief, when antioxidant scavengers of free radicals are added to an active oscillating BR mixture, there is an immediate quenching of the oscillations, an inhibition time (t_inhib_) which depends linearly on the concentration of the antioxidant added, and subsequent regeneration of oscillations. Relative antioxidant activity (r.a.c.) with respect to resorcinol as a standard [33] is determined on the basis of the inhibition times and is defined as the ratio:

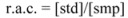

where [smp] is the concentration of the sample added to the mixture giving a certain inhibition time and [std] is the concentration of the standard which give the same inhibition time. This latter concentration is obtained from the straight line equation of the resorcinol; r.a.c. is expressed as μg/mL resorcinol equivalents [19].

#### 3.5.3. TEAC (Trolox Equivalent Antioxidant Capacity) Assay

We used the protocol suggested by Re *et al.* [34]. The green ABTS^•^^+^ radical cation was prepared by mixing ABTS aqueous stock solution (7 mM) with potassium persulphate (2.45 mM). The mixture was kept in the dark until the reaction was complete and absorbance at 734 nm stable. A sample of the *A. spectabilis* extract was dissolved in distilled water. For the photometric assay, 3.0 mL of diluted ABTS^•^^+^ solution and 30 µL of sample were mixed in a photometric cuvette and the absorbance was measured at 734 nm. A blank with distilled water was measured in the same way. The difference between the absorbances of the blank and the sample gave ΔE6 (E6_blank_ − E6_sample_ = ΔE6). For the ME extract, five or six dilutions were measured in triplicate, obtaining a straight line. A stock solution of Trolox, 0.25 mg/mL, was prepared and diluted to an amount ranging from 0.05 to 0.1875 mg/mL. The absorbances of five or six dilutions were measured in the same way as for the sample. The relative antioxidant activity is the ratio:

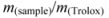

where the *m*s are the slopes of the straight lines, expressed as millimol/L Trolox equivalents [19].

### 3.6. Determination of Total Phenols (Total Reducing Power Quantification)

This test is based on oxidation of phenol groups by phosphomolybdic and phosphotungstic acids (FC reagent). After oxidation, the absorbance of a green-blue complex is measured at 765 nm. We used the procedure for 20 mL total volume of the reacting mixture [35]: a suitably diluted sample solution (2.0 mL) was mixed with FC reagent (10 mL, diluted 1:10 with distilled water) in a 20-mL volumetric flask, and allowed to stand at 24 °C for exactly 5 min. Then a sodium carbonate solution (8 mL) containing anhydrous Na_2_CO_3_ (0.6 g) was added to the mixture. After 2 h at 24 °C, absorbance was measured at 765 nm. The blank (2.0 mL of distilled water) was treated in the same way. For the ME extract, five or six dilutions were measured in triplicate, obtaining a straight line. Gallic acid was used as a standard, and the total reducing capacity or total phenol content is expressed as Gallic Acid Equivalents (GAE) in μg GA per g dry weight extract. 

### 3.7. Iron (II) Chelating Capacity

The ferrous ion-chelating capacity was measured by the methods previously described [24,36]. The extracts were diluted 10-fold with ethanol, then the solution obtained (0.9 mL) was added to 2 mM FeCl_2_ (60 µL), and the reaction mixture was activated by the addition of 5 mM ferrozine (120 µL). After vortexing, the reaction mixture was incubated at room temperature for 10 min and its chelating activity spectrophotometrically measured at 562 nm.

## 4. Conclusions

The present results indicate that proantocxyanidin-rich *A. spectabilis* bark extract has a strong *in vitro* antioxidant effect. The results from the phytochemical investigations are in agreement with DPPH, BR and FC tests. Polyphenols are mainly observed in the ME extract and the antioxidant activity of proantocyanidins and related compounds are well known. Furthermore the compound (**3**) that is present in CH extract do not possess a cathecol group and this is related with poor antioxidant properties [37,38]. Further studies are needed to investigate the *in vivo* pharmacological properties of *A. spectabilis* bark extract, because its high activity may mean that it could be considered as a new antioxidant ingredient for the nutraceutical or functional food market.

## Supplementary Materials

Supplementary materials can be accessed at: http://www.mdpi.com/1420-3049/17/2/1686/s1.
