# Supplementary material for: Phytochemical and Antioxidant-Related Investigations on Bark of Abies spectabilis (D. Don) Spach. from Nepal
Source: Molecules. 2012 Feb 8;17(2):1686–97. doi: 10.3390/molecules17021686 (PMC6268527; doi:10.3390/molecules17021686)
Supplement: Supplementary file 1 [file molecules-17-01686-s001.docx]

*Supporting Information*

**Figure S1.** ^1^H-NMR spectrum of methanol extract of *A. spectabilis* bark.

**Figure S2.** HSQC dept spectrum of methanol extract of *A. spectabilis* bark.

**Figure S3.** ^1^H-NMR spectrum of dichloromethane extract of *A. spectabilis* bark.

**Figure S4a.** Product ion spectra of proanthocyanidin dimer ion at *m/z* 609.

**Figure S4b.** Fragmentation pathway of a proanthocyanidin dimer in *A. spectabilis* bark. The fragment mechanisms are RDA (retro-Diels–Alder), HRF (heterocyclic ring fission).

**Figure S4c.** Product ion spectra of proanthocyanidin dimer ion at *m/z* 593.

**Figure S4d.** Fragmentation pathway of a proanthocyanidin dimer in *A. spectabilis* bark. The fragment mechanisms are RDA (retro-Diels–Alder), HRF (heterocyclic ring fission).

**Figure S4e.** Product ion spectra of proanthocyanidin trimer ion at *m/z* 913.

**Figure S4f.** Fragmentation pathway of a proanthocyanidin trimer in *A. spectabilis* bark. The fragment mechanisms are RDA (retro-Diels–Alder), HRF (heterocyclic ring fission).

**Figure S5.** HPLC-DAD chromatogram at 280 nm of *A.spectabilis* bark methanol extract. The inlet reports the UV spectra relative to the peaks corresponding to proantocyanidin.
